# Irony and Proverb Comprehension in Schizophrenia: Do Female Patients “Dislike” Ironic Remarks?

**DOI:** 10.1155/2014/841086

**Published:** 2014-06-03

**Authors:** Alexander M. Rapp, Karin Langohr, Dorothee E. Mutschler, Barbara Wild

**Affiliations:** Department of Psychiatry, University of Tuebingen, Calwerstraße 14, 72076 Tuebingen, Germany

## Abstract

Difficulties in understanding irony and sarcasm are part of the social cognition deficits in patients with schizophrenia. A number of studies have reported higher error rates during comprehension in patients with schizophrenia. However, the relationships of these impairments to schizotypal personality traits and other language deficits, such as the comprehension of proverbs, are unclear. We investigated irony and proverb comprehension in an all-female sample of 20 schizophrenia patients and 27 matched controls. Subjects indicated if a statement was intended to be ironic, literal, or meaningless and furthermore rated the meanness and funniness of the stimuli and certainty of their decision. Patients made significantly more errors than controls did. Globally, there were no overall differences in the ratings. However, patients rated the subgroup of stimuli with answers given incorrectly as having significantly less meanness and in case of an error indicated a significantly higher certainty than controls. Across all of the study participants, performances in irony (*r* = −0.51) and proverb (*r* = 0.56) comprehension were significantly correlated with schizotypal personality traits, suggesting a continuum of nonliteral language understanding. Because irony is so frequent in everyday conversations, this makes irony an especially promising candidate for social cognition training in schizophrenia.

## 1. Background

Defective appraisal of the intention of others and difficulties with language are hallmark features of psychopathology in schizophrenia. Social cognition deficits have been previously identified and are currently being extensively researched. The results show that deficits in social cognition are relevant to real-world functioning and outcome [1]. Training of social cognitive skills has gained increasing interest in schizophrenia therapy. In this context, the comprehension of ironic remarks by patients with schizophrenia has become a research focus both as an outcome measure and as a training goal [1–5].

This is obvious, considering that the decision on whether a remark made by others is intended to be ironic or not is slightly artificial, but is instead required routinely in everyday interaction. Everyone is familiar with irony. It is remarkably frequently used, as shown by linguistic research [6, 7]. For example, Gibbs [8] showed that 1 out of 8 conversational turns in an everyday communication among college students was ironic. In the case of linguistic irony (which alone is discussed here), an ironic expression is usually incorrect and often the opposite of what the speaker intends to communicate [9]. Perhaps even more relevant to social cognition research is that irony serves important communicative functions [10, 11], such as expressing negative emotional content [12, 13], expressing anger [14, 15], creating an impression of being a humorous person [8, 16], disarming a difficult conversational situation [12, 17, 18], or—in the case of sarcasm—meaning the opposite and mocking someone. The fact that irony is more often used in difficult stages of communication [9, 16, 19] is also relevant in the context of schizophrenic impairment. Since ironic remarks are ambiguous per se, irony comprehension also poses challenges to ambiguity resolution [20, 21], which is a cognitive operation patients with schizophrenia are known to have difficulties with [22–24].

Previous research has shown that patients with schizophrenia, as a group, have difficulties in irony comprehension [3, 25–29], but not all patients show a deficit [30, 31]. However, given the increasing interest in irony for social cognition training and assessment, more profound knowledge of the difficulties faced by patients with schizophrenia in irony comprehension seems worthwhile [1]. Many studies on irony in schizophrenia are motivated by the intention to provide measurements of theory of mind (TOM) and perspective taking deficits [2, 5, 26] in schizophrenia because irony comprehension, essentially, requires building of a second-order TOM [32–34].

However, defective irony comprehension in schizophrenia is likewise influenced by numerous additional cognitive operations that individuals with schizophrenia are known to have difficulties with. For example, irony also relates to higher-level language comprehension [10, 34–37]. Traditionally [38–40], and as documented by a large body of research [41–45], schizophrenia is considered to lead to a deficit in comprehension of nonliteral language. Nonliteral language, such as proverbs, metaphors, and idioms, is interpreted “word by word” (literally) as either wrong or wrong in a particular context. The same applies to irony and sarcasm, because in many or even most cases, ironic statements taken literally state the exact opposite of what is intended (e.g., the remark “oh brilliant!” when something bad happens). Irony and sarcasm are therefore important subtypes of nonliteral language. Traditionally, it has been assumed that patients with schizophrenia show a cognitive bias towards literal interpretation of nonliteral expressions [46, 47]. In the case of irony, that would result in assuming the exact opposite of the intention. However, only limited research [2, 5, 25, 26] has been conducted on the relationship between irony comprehension and bias towards literal interpretation of other stimuli, such as metaphors, and even these studies have often used only small numbers of stimuli.

Another interesting point that has not received much attention in past research is how patients with schizophrenia appreciate ironic remarks. Most previous studies have addressed performance (i.e., whether patients fail to comprehend ironic remarks), while it is widely unknown if patients with schizophrenia “like” ironic remarks or they perhaps judge their intention as more mean than healthy controls would do. This relationship to the perceived “meanness” by patients with schizophrenia seems particularly interesting, because judging the intention of others as “mean” or “hostile” may relate to the origin of delusions in these patients [48, 49]. Indeed, it has been suggested (e.g., by [21, 50]) that misinterpretation of “ambiguous and hard-to-interpret communicative signals” such as “ironic comments” in combination with the above-mentioned factors may contribute to delusion formation in schizophrenia. Ironic comments are often intended to be funny [8, 11, 16]. Patients with schizophrenia showed aberrant ratings for the jocularity of stimuli in previous investigations [51–55]; however, to our knowledge, no research has addressed the funniness of ironic comments in schizophrenia.

There is consensus in the literature that the severity of irony comprehension deficit differs between individuals with schizophrenia. However, it is currently unclear whether this deficit depends on a continuum with schizophrenia and what the possible mediating factors could be. One possible mediating factor for deficits in sarcasm comprehension that has been discussed, but not consistently found, in the literature is schizotypal personality traits [35, 56–58]. A continuum is assumed for schizotypal personality traits between schizotypal traits in nonclinical subjects and symptoms manifested in patients with schizophrenia [21, 59, 60]. However, no studies have investigated irony comprehension in relation to schizotypal traits in both patients with schizophrenia and controls in a single study.

Our pilot study aimed to investigate both comprehension and appreciation of ironic remarks in a sample of patients with schizophrenia. On the one hand, we aimed to investigate comprehension and whether it was related to comprehension of proverbs, the most often applied measurement of concretism in schizophrenia. We applied an irony comprehension task recently developed by our group [35, 58]. In this task, which is a written task that is prosody-free, subjects decide by button press whether the statement of a protagonist in short text vignettes is intended, literal, ironic, or meaningless. In addition, the subjects rated their self-certainty for that decision in each stimulus and rated the “meanness” and “funniness” of the statements on a Likert scale.

We have several hypotheses concerning the results. Since lower performance in irony detection is documented in several previous investigations [25–29], our hypothesis was that patients may find the decision if a conversational remark is intended to be ironic more difficult and, therefore, would show lower certainty in their decisions, especially for ironic stimuli. We further assumed the supposed higher-level difficulty of the decision about their intention will result in schizophrenic patients judging the stimuli as more mean and less funny. Following the continuum hypothesis [60] and based on our previous functional magnetic resonance imaging findings [21, 35] we expected to find an association between schizotypal personality traits and performance across all subjects irrespective of the presence or absence of a schizophrenia diagnosis.

## 2. Materials and Methods

### 2.1. Subjects

20 right-handed [61] individuals with DSM IV schizophrenia and 27 right-handed healthy control subjects gave written informed consent and participated in the study. All study subjects were female. Control subjects were recruited from the general population and were free from psychiatric illness. Groups were matched for age, educational level, and verbal intelligence (see Table 1).

Patients were recruited from the Department of Psychiatry, University of Tübingen, Germany. Patients were acute or subacute inpatients and predominantly of the paranoid subtype. All participants were native German speakers, had no other past or present medical illness, and had sufficient reading skills. A subgroup of the sample additionally participated in a functional magnetic resonance imaging study [21]. All patients were on stable medication, mainly with atypical antipsychotics (mean dosis [65] 534 (SD: 230) chlorpromazine equivalents).

### 2.2. Procedure

First, all subjects received complete information about the study and ability to consent was ensured. Then, subjects underwent a practice session for the irony comprehension task with stimuli not used in the experiment and provided written informed consent. The study was approved by the local ethical committee (University of Tuebingen, Germany). Subjects then completed the irony comprehension task. The sequence of the other tests was counterbalanced between the subjects. If requested by the subject, a short break between the tests was possible.

### 2.3. Irony Comprehension Task

An irony comprehension and evaluation task was applied to all subjects. Stimuli and stimulus sequence are identical with a previous functional magnetic resonance imaging study [21, 35] from our group. However, comprehension assessment was modified. In addition, for each target sentence, subjects rated certainty of their decision, meanness of the statements, and jocularity of the statements.

The irony comprehension task consists of 56 short German text vignettes as stimuli, each consisting of an introductive context (2 sentences, 8–12 words) and two or more protagonists (e.g., “Petra hates fish. Her mother has cooked salmon for her.”). The introductive context is then followed by a target part, in which one of the protagonists makes a statement (e.g., “Petra says: ‘Oh brilliant, my favourite meal!'”). This statement has, in this context, either an ironic or a literal meaning or is meaningless.

The number of words and sentences, grammatical complexity, and word frequency are counterbalanced between literal, ironic, and meaningless context scenarios.

Corresponding literal and ironic target sentences are identical. For example, for the above ironic stimulus, the literal counterpart would be, “Katja loves spaghetti. Her mother has cooked a lot of spaghetti for her. Katja says: ‘Oh brilliant, my favourite meal!”'

A comprehensive description of the stimuli and their development and evaluation process is given in [35, 58].

During the irony task, subjects were seated in front of a notebook computer (Dell Inspiron) next to one of the investigators (K.L.). In-house software was used to present the stimuli and measure the button press response. Stimuli were presented in a pseudorandomized order not foreseeable for the subject. The introductive context was both presented visually and read out by the investigator. To avoid any effect of prosody, target sentences were only presented visually. The task was to indicate by pressing one out of three buttons whether the target sentence was in this context most likely ironic, literally meaningful, or meaningless. After each stimulus and button press, subjects were asked by the investigator (K.L.) to rate the certainty of their decision on a 5-point Likert scale (from 0, very uncertain, to 4, very certain) as well as the meanness (from 0, not mean at all, to 4, very hurtful) and jocularity (from 0, not funny at all, to 4, very funny) of the protagonist's statement.

### 2.4. Proverb Comprehension Test

A multiple-choice German proverb test developed at the University of Münster [66, 67] was administered to all participants. It is a paper and pencil test without a time limit. This test has a higher than average number of test stimuli, as it consists of 32 salient German proverbs/idioms. A comprehensive description of the test and its development is given in Thoma et al. [66] and Uekermann et al. [67]. Some test proverbs/idiom stimuli are literally true, whereas others are not. The correct meaning cannot always be determined if a subject is unaware of the correct meaning. A major advantage of the test is that it enables an analysis of performance with respect to the subjects' familiarity. It consists of two procedures. First, the subject rates how familiar he or she is with the proverb given on a 5-point Likert scale (from 1, never, to 5, a lot of times). In a second procedure, the subject is presented with the proverbs again and asked to decide (multiple choice) which out of the 4 given answers best explains the meaning. Type I represents a definition that is based on the one given by the German dictionary of proverbs [68] and is subsequently referred to as the correct one. Type II (abstract/meaningless), Type III (concrete meaningful), and Type IV (concrete/meaningless) represent distractors.

Several scores were defined in our investigation. First a proverb familiarity score was calculated for each individual. It consists of the mean values of the familiarity ratings of all 32 proverbs. Secondly, mean values for numbers of proverbs rated as unfamiliar were compared between patients and controls. Here, we defined a proverb as “familiar” if its rating is above 1; that means it is ready counted as familiar if the subject indicates he has heard it once before. Each Type I answer given is counted as one point, whereas all other answers in multiple choice are counted zero. This results in a sum score for each participant. The higher the score is, the better the performance is. The highest reachable sum score is 32. All these scores were compared between the groups.

### 2.5. Other Assessment

After completion of the previous tests and the schizotypal personality questionnaire [69], German version by [64], a verbal intelligence test (Mehrfachwahl-Wortschatz test version B; [63]) and the subtest “picture sequencing” from the HAWIE-R intelligence test [62] were applied.

In addition, patients were interviewed by a psychiatrist (A.R.) to evaluate current psychopathology using the SAPS [70], SANS [71], and PANSS [72] and to evaluate functioning (GAF).

### 2.6. Statistical Analyses

SPSS 11.5 was used for all statistical analyses. Due to the exploratory character of the study and the small sample size, a significance level of 0.05 was chosen.

## 3. Results

### 3.1. Irony Comprehension

**Figure 1:**
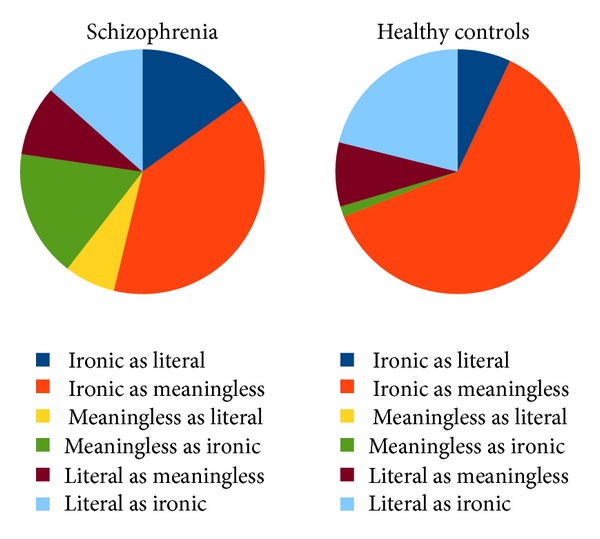
Pattern of errors in the irony comprehension test of patients (left) and control subjects (right).

*Performance*. Regarding the percentage of correct answers, there was a significant difference between schizophrenia patients and healthy control subjects (*P* < 0.001, ANOVA): while the healthy control group had 95% correct answers on average, patients had only 85% correct answers. The error profile was also different between patients and controls (see Figure 1) (*P* = 0.09; Mann-Whitney *U* test).

### 3.2. Judgement/Appreciation of Ironic Remarks

For the whole task with all of the stimuli taken into account, the patient and control groups did not differ in their certainty, in their judgement of meanness, or in how funny they rated the stimuli (Table 2). The stimuli were then analysed in further detail. All of these results are shown in Table 2. First, the types of stimuli were analysed separately for ironic, literal, and nonsense stimuli. Neither the ratings for ironic nor those for literal sentences differed significantly. There was only a significant difference for the meaningless stimuli in funniness judgement in that patients with schizophrenia rated the meaningless sentences significantly more funny (*P* = 0.002).

**Figure 2:**
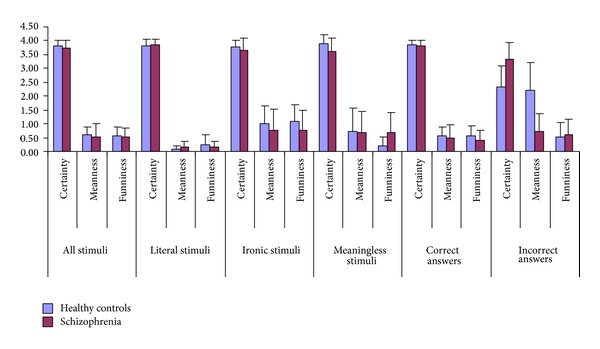
Detection and appreciation of irony. Group differences.

In a next step, the ratings for answers given correctly and the answers given incorrectly were compared between the groups. Control subjects and patients with schizophrenia reported significantly lower certainty for the stimuli that they responded to with an error compared to those to which they replied correctly (*P* = 0.004 in patients with schizophrenia; *P* < 0.0001 in controls). However, the decline in perception was more enhanced in control subjects relative to patients (see Figure 2). Stimuli that they responded to with an error were rated as significantly less certain by controls than by patients.

In the case of meanness ratings, control subjects (*P* > 0.0001), but not patients with schizophrenia (*P* = 0.26), showed an increase in rated meanness for stimuli they responded to with an incorrect judgement as compared to ones they answered correctly.

There were no differences between correct and incorrect stimuli in either group for perceived funniness.

### 3.3. Association of Schizotypal Personality Traits with Performance

**Figure 3:**
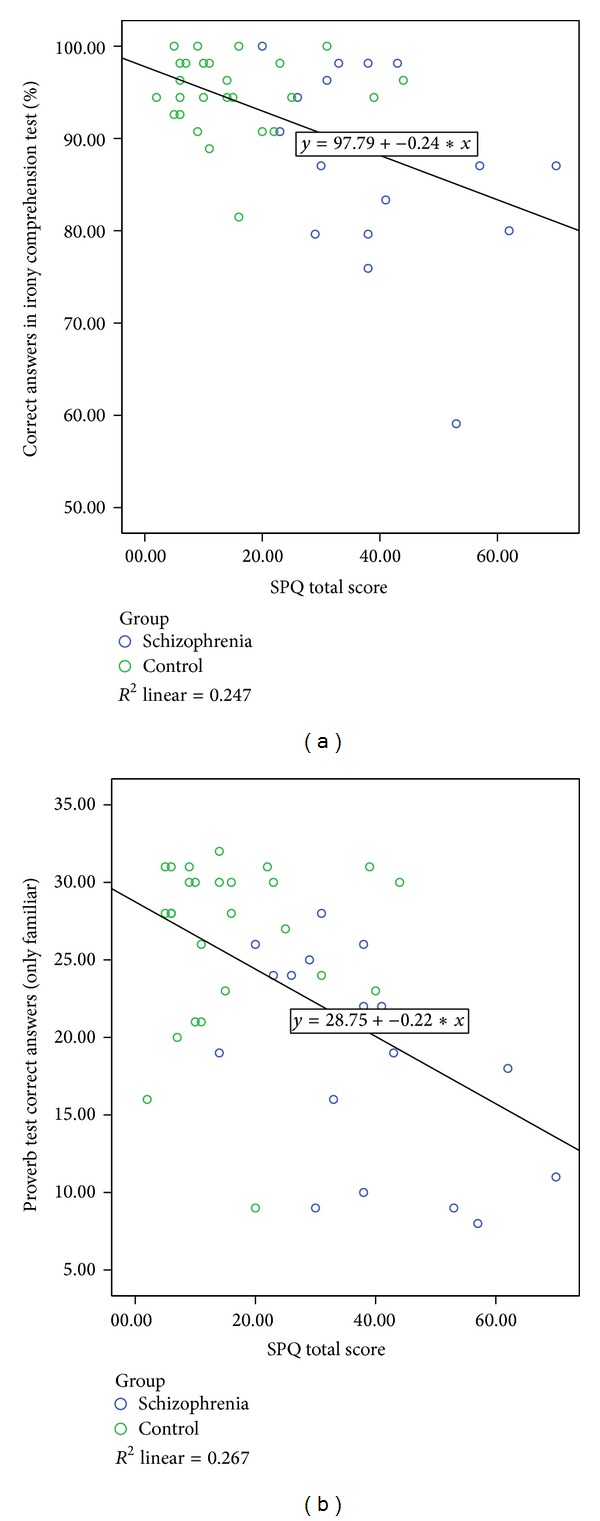
Correlation between performance in the (a) irony comprehension test and (b) proverb comprehension test and schizotypal personality traits.

This analysis was calculated for all participants together, following the rationale that schizotypal traits represent a continuum [59]. There was a significant correlation between the performance in the irony comprehension test (% correct answers) and the total score of the SPQ (PEARSON  *r* = −0.51; *P* < 0.0001) (Figure 3(a)). However, there was no correlation between the SPQ and the rating measures. There was also a significant correlation between proverb test performance and the SPQ total score (familiar stimuli *r* = −0.46; *P* = 0.002 (Figure 3(b)); all proverbs *r* = −0.56; *P* = 0.00009).

## 4. Discussion

We investigated the comprehension and appraisal of higher-level language in female patients with schizophrenia and matched healthy control subjects. Subjects completed a German irony comprehension and appraisal test [58], which consists of text vignettes that end with a statement of one of its protagonists and this statement is (in this context) either ironic, literal, or meaningless. Congruent with our expectation, patients made significantly more errors in this test, as a group and relative to healthy controls.

### 4.1. Interpretation Ratings

Beyond investigating performance, a main goal of the study was to understand how patients with schizophrenia would evaluate and appreciate ironic remarks. As outlined in the introduction, we hypothesized that patients with schizophrenia would be less certain in their decision about intention and would indicate a higher degree of meanness for the stimuli, especially those intended to be ironic, and would rate them less funny. None of these hypotheses was clearly confirmed. Only meaningless stimuli were rated significantly higher in their funniness, a finding that is compatible with previous findings of differences in funniness ratings in patients with schizophrenia [55]. However, it must be noted that this was contrary to our hypothesis. Perhaps even more relevant was the finding that the overall difference was small and there was little variance. The data about certainty was less interesting, because although patients with schizophrenia made significantly more errors, they indicated equal certainty of their decisions. Since 85% of the correct answers could still be regarded as a positive performance, evaluation of irony could be impaired only to a small degree in schizophrenia. Therefore, it is possible that our sample size was insufficient to detect a significant effect. However, another possibility is that patients had difficulties with our metacognitive evaluation task since one of the instructions was to reflect about “self-certainty of their decision.” Schizophrenia, however, may go along with a fundamental disturbance of self-certainty [73], and that may have altered ratings in our task. For example, delusions, doubtlessly one of the hallmark symptoms of schizophrenia, are unshiftable false beliefs and represent perturbance of certainty of an interpretation [74]. The pattern of response for certainty ratings for items judged wrong by the individual is compatible with such an effect while there is no significant difference in ratings between controls and patients with schizophrenia for items where performance was correct. There was a highly significant difference in items judged wrong; patients with schizophrenia were more certain in their (wrong) decisions, which is in line with previous findings and historical reports of a higher degree of unconcern in semantic judgment in schizophrenia. Moreover, they rated stimuli judged false as less mean. This may be interpreted as unconcern towards irony. However, our original hypothesis that female patients with schizophrenia may “dislike” ironic remarks is not supported.

### 4.2. Proverb Comprehension

An additional aspect to our investigation was the evaluation of performance in the multiple-choice proverbs test. As expected, we found an impairment in schizophrenia. Our finding of an impairment of proverb comprehension in schizophrenia mirrors numerous classical findings [40, 41, 45]. For example, similar to this study, a seminal investigation by Elmore and Gorham [75] found severe impairments in female patients with schizophrenia when they had to match proverbs with the appropriate alternative meaning in a multiple-choice test, a finding that is confirmed by a substantial number of studies [45]. However, research from both healthy and clinical populations indicates familiarity is a factor that critically interrelates with proverb test performance [37]. Familiar proverbs (that mean proverbs that the individual “knows”) are much easier to interpret [45, 67]. Of note, this factor was not recommended in many previous investigations in schizophrenia; however our results indicate it is relevant: the impairment in schizophrenia was more pronounced when we analyzed only the stimuli that the subject said she was familiar with. This result was in line with the findings from the test developers of the German proverb test used here [66], which showed that patients with schizophrenia in a mixed gender sample are impaired in the test. However, in contrast to the investigation by Thoma et al. [66], in our sample, the difference in familiarity was not significant. Nevertheless, our results further underline the importance of familiarity for proverb comprehension described by this study.

### 4.3. A Possible Role of Schizotypal Personality Traits

Irrespective of the diagnosis when means were correlated across all study participants (patients and controls), the performance was correlated with schizotypal personality traits: the higher the total score of the schizotypal personality questionnaire (SPQ), the lower the performance in either the irony or proverb test. This result is in line with increasing evidence suggesting that schizotypal personality traits represent a continuum from healthiness to schizophrenia [59, 76] and, in a broader sense, our results further strengthen the increasing evidence of a continuum between healthiness and psychosis/schizophrenia. It has repeatedly been suggested that such a continuum might be present for language symptoms; however, our study is the first to demonstrate that, for nonliteral expressions, it is present for proverbs and ironic remarks. On the basis of our results, we conclude that schizotypal personality traits could possibly mediate nonliteral language impairment in schizophrenia. A possible cerebral correlate is a finding that brain activation in the medial prefrontal lobe/dorsal ACC during comprehension was associated with comprehension of ironic but not literal statements in our task in a recent fMRI study from our group [21]. It is important to note, however, that the patient sample from this study has overlap with our investigation.

Another limitation is that patients and control subjects significantly differ in their expression of schizotypal personality traits, although Figure 3 shows there is notable overlap between the groups. This limits the generalizability of our findings and makes future studies with a broader range of schizotypal symptoms in their nonclinical samples eligible, especially since the significant correlation between SPQ and proverb and irony comprehension performance reported here is in contrast to other studies with only nonclinical populations that failed to find such an association. For example, Langdon and Coltheart [56] found no association between irony and metaphor comprehension and psychometric schizotypy. In another nonclinical study [57], irony comprehension was assessed using the awareness of social inference test (TASIT); [77]; they also found no difference between high and low schizotypy. Using the same irony task as ours in a different sample of 15 healthy female subjects, Mutschler [58] found no performance difference between high and low expression of schizotypy. For other types of nonliteral stimuli, Humphrey et al. [78] failed to find a difference between high and low SPQ individuals on metaphor comprehension in a larger sample from New Zealand. However, compared to our study, all these investigations included a smaller range than our sample.

## 5. Limitations

We are aware of several limitations in our study. Several of them are related to the characteristics of our irony comprehension task [21, 35]. First, some relate to the characteristics of the stimulus material. Our stimulus material consisted of stimuli that were matched and counterbalanced for complexity, grammar, and word frequency. They were written material and therefore free from prosody and other social information like gesture and facial expressions. This may represent both a strength and a limitation of the study. Almost all factors mentioned above have been described to influence the decision whether intention is ironic or literal in previous studies and, importantly, each of them has been found impaired in schizophrenia previously. Our study makes clear that difficulties exist in schizophrenia even when prosody is eliminated and social context information reduced. However, our pilot study cannot answer whether adding such information improves irony comprehension in schizophrenia, so that studies systematically varying these factors become eligible. Current literature cannot answer these questions either. Studies differ in their amount of contextual and social cues: some are, like ours, prosody-free [79]; others used video clips, including gesture and prosody [27–29, 80]; and others even specifically addressed the effect of tone of voice [81]. However, these studies vary in schizophrenia disease severity and other factors as well. In our ironic text vignettes, protagonists made ironic comments towards another protagonist or about the environment. However, the reaction of patients with schizophrenia may be different when ironic statements are made towards the individual directly. In contrast to a real-world environment, where irony is not explicitly marked, subjects were informed that irony may occur during the stimuli and this priming may make the task less difficult. Affective connotation is a further limitation. Like those in most previous investigations, ironic and literal stimuli are not entirely matched for affective connotation in that ironic stimuli are more often negative than literal counterparts. It has been suggested that irony with positive connotations may be more difficult to comprehend, so that future investigations should investigate irony with positive connotation in patients with schizophrenia.

Sample size is another limitation of this study. Although it was within the range of comparable investigations [2, 5, 79, 81], the sample size is small given the heterogeneity of the disorder and gives our investigation the character of a pilot study.

In this study, we found a significant association of schizotypal personality traits with performance. However, SPQ values were significantly higher in patients with schizophrenia, and in our pilot study, we did not control for other personality variables. For example, other personality traits not measured here may be associated with irony comprehension as well [11, 82].

Another limiting factor in our study is gender. To enhance homogeneity of the sample, we investigated only female subjects. This point is relevant since gender differences have been reported for irony appreciation in healthy subjects [11, 83], in schizophrenia [84], and for schizotypal personality traits [85].

## 6. Conclusions/Implications

We see several implications from our study. Similar to a previous study [31], our study made it clear that the irony comprehension deficit in schizophrenia is not restricted to the “classical” symptom named concretism that claims a tendency towards the literal interpretations in schizophrenia. Instead, the error pattern also includes difficulties in disentangling the opposite direction, as a significant proportion of errors made by patients with schizophrenia are in misjudging the intention of meaningless and literal statements as ironic. Future schizophrenia research in the fields of pragmatic language comprehension and research on (mis)interpretation of the intention of others are warranted. In our pilot study, we cannot, due to the small sample size, disentangle which psychopathology dimensions are interrelated with this deficit. Positive symptoms, especially persecutory delusions, seem especially interesting in this context, as the relevance of capturing the intention (in this case linguistic) of others is impaired in these conditions, and the relationship between discriminating literal and ironic intention seems, therefore, a straightforward paradigm [21, 28, 32]. However, other schizophrenic symptomatologies, including cognitive deficits [30], altered theory of mind and perspective taking [2, 29], disorganized symptoms, negative symptoms [31, 79, 86], and formal thought disorder [31, 87], may be interrelated with irony comprehension as well.

Despite these limitations, we were able to demonstrate that female patients with schizophrenia are impaired in irony comprehension and show reduced insight and aberrant response, especially to those stimuli judged wrong. This, together with the observation that irony is so remarkably frequent in everyday conversations and is even more frequent in difficult communicative situations, which per se have a higher ambiguity, makes irony an especially promising candidate for social cognition training in patients.

## Figures and Tables

**Table 1:** Clinical and sociodemographic characteristics of patient and control groups.

	Healthy controls	Schizophrenia	Significance
	*N* = 27	*N* = 20	
Age	38,9	34,1	n.s.
Education years^1^	15,1	14,2	n.s.
Digit span (digits)	6,2	6,1	n.s.
CPT errors	0,1	1,5	0.0026
Verbal intelligence^3^	31,3	30,6	n.s.
HAWIE picture sequencing test^2^	32,5	26,4	n.s.
SPQ cognitive perceptual^4^	10,1	22,3	0.00007
SPQ interpersonal^4^	5,7	14,8	0.00005
SPQ total score	15,6	36,7	0.00000
PANSS total score		70,3	
SANS total score		36,1	
SAPS total score		35,7	
Global assessment of functioning scale		37,9	

^1^In full-time education.

^2^Subtest 2 from [62].

^3^Multiple-choice vocabulary test [63].

^4^As defined by [64].

**Table 2:** Group differences in detection and appreciation of irony.

		Healthy controls	Schizophrenia	Significance
All stimuli	Certainty	3,81	3,73	0.26
	Meanness	0,58	0,52	0.58
	Funniness	0,57	0,51	0.54

Literal stimuli	Certainty	3,81	3,85	0.59
	Meanness	0,09	0,15	0.26
	Funniness	0,22	0,15	0.43

Ironic stimuli	Certainty	3,79	3,67	0.21
	Meanness	1,02	0,77	0.24
	Funniness	1,09	0,76	0.10

Meaningless stimuli	Certainty	3,88	3,60	**0.02**
	Meanness	0,70	0,69	0.95
	Funniness	0,19	0,70	0.002

Correct answers	Certainty	3,84	3,82	0.70
	Meanness	0,56	0,49	0.55
	Funniness	0,57	0,41	0.16

Incorrect answers	Certainty	2,34	3,34	**0.00002**
	Meanness	2,23	0,72	**0.000001**
	Funniness	0,52	0,60	0.64
